# Exposure of *Aspergillus fumigatus* to Atorvastatin Leads to Altered Membrane Permeability and Induction of an Oxidative Stress Response

**DOI:** 10.3390/jof6020042

**Published:** 2020-03-26

**Authors:** Ahmad Ajdidi, Gerard Sheehan, Kevin Kavanagh

**Affiliations:** Department of Biology, Maynooth University, Maynooth, Co. Kildare W23F2K8, Ireland; ahmad.ajdidi.2013@mumail.ie (A.A.); gerard.sheehan.2013@mumail.ie (G.S.)

**Keywords:** atorvastatin, *Aspergillus*, ergosterol, infection, proteomics

## Abstract

*Aspergillus fumigatus* is a serious cause of disease in immune-deficient patients and in those with pulmonary malfunction (e.g., cystic fibrosis (CF), asthma). Atorvastatin is a member of the statin drug family, which are the main therapeutic agents used to decrease high serum cholesterol levels by inhibiting (HMG-CoA) reductase enzyme. The aim of the work presented here was to analyse the antifungal activity of atorvastatin and assess its effect on the virulence of *A. fumigatus*. Atorvastatin demonstrated strong antifungal activity and reduced the growth and viability of *A. fumigatus*. Exposure of *A. fumigatus* to atorvastatin led to a reduction in ergosterol content and increased membrane permeability, as evidenced by the release of protein, amino acids and gliotoxin. Proteomic analysis revealed an increased abundance of proteins associated with an oxidative stress response, such as the glutathione s-transferase family protein (+8.43-fold), heat shock protein Hsp30/Hsp42 (+2.02-fold) and 5-demethoxyubiquinone hydroxylase, mitochondrial (+1.73-fold), as well as secondary metabolites such as isocyanide synthase A icsA (+8.52-fold) and non-ribosomal peptide synthetase fmpE (+3.06-fold). The results presented here indicate that atorvastatin has strong antifungal properties and may have potential application in the treatment of *A. fumigatus* infections alone or in combination with existing antifungal agents.

## 1. Introduction

*Aspergillus fumigatus* is a serious cause of disease in immunocompromised patients or in those with pulmonary malfunction. *A. fumigatus* is widely distributed in the environment and produces large numbers of conidia [1,2], which are inhaled on a daily basis. A rapid immune response eliminates these in the immunocompetent host but in cases of pulmonary disease (e.g., cystic fibrosis (CF), asthma) or immunosuppression conidia can quickly germinate and grow in the lung [1,3]. The fungus can cause three types of disease. Saprophytic infection involves the growth of the fungus in the lung and is seen in asthmatic and CF patients [4]. Allergic bronchopulmonary aspergillosis occurs when the body reacts to toxins and allergens secreted by the fungus as it grows in the thick mucus secretion found in the CF or asthmatic lung. Invasive aspergillosis occurs in immunocompromised patients and involves the fungus growing through viable tissue and disseminating to other sites in the body [5]. Invasive aspergillosis can have a mortality rate of 80%–90% and is difficult to diagnose and treat [6]

*A. fumigatus* displays a number of characteristics that facilitate its growth and persistence in the lung. The fungus is thermotolerant and can survive temperatures up to 45 °C [7]. It is capable of producing a wide range of enzymes that may facilitate the degradation of tissue and toxins (e.g., gliotoxin, fumagillin), which may play a role in suppressing the local immune response [8,9].

Treatment of aspergillosis involves the use of a range of antifungal drugs. Azole antifungal agents, such as voriconazole, show efficacy against invasive aspergillosis. The polyene Amphotericin B has been widely used for the treatment of invasive aspergillosis and, due to its inherent toxicity, is now usually administered in a liposomal formulation [10,11]. Echinocandins, such as caspofungin, show good efficacy against aspergillosis and are well tolerated in vivo [12].

Statins are widely used for the control of cholesterol and act by inhibiting the action of 3-hydroxy-3-methylglutaryl-CoA (HMG-Co A) reductase and thus block cholesterol biosynthesis [13,14]. Statins may be produced naturally (e.g., lovastatin), semi-synthetically (e.g., simvastatin) or synthetically (e.g., atorvastatin) and were originally obtained from a variety of fungi (e.g., *Aspergillus terreus, Penicillium citrinum*) [14]. Mammalian cholesterol and fungal ergosterol are structurally similar and their biosynthetic pathways are closely related [14]. Consequently, fungal cells exposed to statins show reduced ergosterol content [15]. Statins have well-established antifungal properties and have been shown to inhibit the growth of a wide range of filamentous fungi (e.g., *A. fumigatus, Aspergillus flavus, Aspergillus terreus, and Aspergillus niger*) [16] and yeasts [15]. Exposure of *Candida glabrata* to simvastatin resulted in inhibition of growth, reduced levels of ergosterol and disruption of mitochondrial DNA [17]. Statins can exhibit fungicidal properties but only at concentrations in excess of those that can be clinically achieved [16]. It has been reported that patients on high doses of statins show fewer fungal infections [18,19,20], prompting the suggestion that statins may represent a well-tolerated antifungal therapy that could be used alone or in combination with conventional therapies for the treatment of recalcitrant infections [21].

The aim of the work presented here was to characterize the response of *A. fumigatus* to one of the most widely prescribed statins, atorvastatin, with a view to determining its potential as a novel antifungal therapy.

## 2. Materials and Methods

### 2.1. Aspergillus Isolates

Conidia of *A. fumigatus* ATCC 26,933 were isolated from malt extract agar (MEA) plates using PBS-Tween (PBS +0.5% (v/v) Tween 80) and harvested by centrifugation (2056× *g*) for 10 min. The conidial pellet was washed twice with 5 mL PBS and resuspended in SAB liquid medium at a concentration of 5 × 10^6^/mL.

### 2.2. Statin

Tablets of atorvastatin (20 mg Film-Coated Rowex Ltd) were crushed to a powder and dissolved in sterile PBS. The solution was filtered sterilised using 0.45 μm and 0.02 μm pore filters.

### 2.3. Susceptibility of A. fumigatus to Atorvastatin

The conidia suspension (100 µL) was added to each well of a 96-well plate containing serial dilutions of atorvastatin in 100 µL of Sabouraud Dextrose Broth. The plate was incubated at 37 °C for 48 h. The optical density at 600nm was determined using a microplate reader (Synergy HT, Bio-Tek) and growth was quantified as a percentage of control.

### 2.4. Effect of Atorvastatin on A. fumigatus on Biomass Production

Cultures *A. fumigatus* were grown on MEA plates for 3–4 days at 37 °C and the conidia were harvested using sterile 0.5% (v/v) Tween 80. The concentration of conidia was adjusted to 5 × 10^6^ conidia/mL in sterile PBS. This conidial suspension was used to inoculate 100 mL Sabouraud dextrose broth supplemented with atorvastatin (1.5 and 6 µg/mL). The culture was incubated at 37 °C in a shaking incubator at 200 rpm for 72 h and the fungal hyphae were weighed after collection by filtering through miracloth (Calbiochem) and exclusion of remaining culture medium.

### 2.5. Ergosterol Extraction and Quantification

Ergosterol was extracted from 1 g *A. fumigatus* mycelium as described in [22]. Ergosterol quantification was performed using a gas chromatograph (Hewlett Packard 5890 series II) with a flame ionization detector and a chrompack capillary column (Chrompack International BV, Middleburg, The Netherlands). The carrier gas was N_2_, and the injector and detector temperatures were set at 320 °C. Ergosterol standards were used to calibrate the instrument.

### 2.6. Determination of Amino Acid and Protein Leakage

*A. fumigatus* was grown for 72 h and exposed to atorvastatin (1.5, 3 and 6 µg/mL) for 2, 4 and 6 h at 37 °C. The release of amino acids was determined using the ninhydrin colorimetric method and expressed in terms of aspartic acid and glutamic acid [23]. Ninhydrin (Sigma-Aldrich) was dissolved in ethanol to give a final concentration of 0.35% (w/v) and 200 μL was added to each sample (1 mL) and heated to 95 °C for 4 min followed by cooling on ice. The absorbance at 600 nm was recorded on a spectrophotometer (Beckman DU 640 Spectrophotometer). The quantity of protein released from the hyphae in the supernatant was assayed using the Bradford assay (Bio-Rad), with bovine serum albumin (BSA) (Sigma-Aldrich) as standard.

### 2.7. Gliotoxin Extraction and Qquantification

Seventy-two hours *A. fumigatus* cultures were harvested by filtration using miracloth. Chloroform (20 mL) were added to an equal volume of each filtrate sample and the solution was mixed continually for 2 h. The chloroform was evaporated to dryness in a rotary evaporator. Dried extracts were dissolved in 500 µL methanol (Fisher chemical) and stored at -20 °C until assayed. Gliotoxin was determined by reverse-phase HPLC (Agilent 1200 Series). The mobile phase was acetonitrile (Fisher chemical), 0.1% (v/v) trifluoroacetic acid (Sigma-Aldrich) and deionized-distilled water. Gliotoxin extract (20 µL) was injected onto a C18 Hewlett Packard column. Gliotoxin concentrations (50, 100 and 200 ng/mL) were dissolved in methanol (Sigma-Aldrich) to create a standard curve of peak area versus gliotoxin concentration.

### 2.8. Protein Extraction and Preparation for Label-Free Mass Spectrometry Analysis

Label-free shotgun semi-quantitative proteomics was conducted on *A fumigatus* hyphae pre-grown in the presence of atorvastatin (3 µg/mL) for 72 hours. Protein was extracted as described by [24]. Briefly, protein (75 μg) was reduced with dithiothreitol (DTT; 200 mM) (Sigma-Aldrich), alkylated with iodoacetamide (IAA; 1 M) (Sigma-Aldrich) and digested with sequence grade trypsin (Promega, Ireland) at a trypsin:protein ratio of 1:40, overnight, at 37 °C. Tryptic peptides were purified for mass spectrometry using C18 spin filters (Medical Supply Company, Ireland) and 1 μg of peptide mix was eluted onto a Q-Exactive (ThermoFisher Scientific, U.S.A) high resolution accurate mass spectrometer connected to a Dionex Ultimate 3000 (RSLCnano) chromatography system. Peptides were separated by an increasing acetonitrile gradient on a Biobasic C18 Picofrit™ column (100 mm length, 75 mm ID), using a 65 min reverse-phase gradient at a flow rate of 250 nL/min. All data were acquired with the mass spectrometer operating in an automatic data-dependent switching mode. A high-resolution MS scan (300–2000 Dalton) was performed using the Orbitrap to select the 15 most intense ions prior to MS/MS. 

### 2.9. Protein Data and Analysis

Protein identification from the MS/MS data was performed using the Andromeda search engine in MaxQuant (version 1.2.2.5; http://maxquant.org/) to correlate the data against the proteome of *A. fumigatus* obtained from Uniport. The following search parameters were used: first search peptide tolerance of 20 ppm and second search peptide tolerance of 4.5 ppm, with carbamidomethylation of the cysteines set as a fixed modification, while oxidation of methionines and acetylation of N-terminals were set as variable modifications and a maximum of 2 missed cleavage sites allowed. False Discovery Rates (FDR) were set to 1% for both peptides and proteins and the FDR was estimated following searches against a target-decoy database. Peptides with minimum length of seven amino acid length were considered for identification and proteins were only considered identified when more than one unique peptide for each protein was observed. Results processing, statistical analyses and graphics generation were conducted using Perseus v. 1.5.5.3. LFQ intensities were log_2_-transformed and ANOVA and t-tests between the proteome of 24-h control and atorvastatin-treated (3 μg/mL) *A fumigatus* was performed using a p-value of 0.05 and significance was determined using FDR correction (Benjamini–Hochberg). Proteins that had non-existent values (indicative of absence or very low abundance in a sample) were also used in statistical analysis of the total differentially expressed group following imputation of the zero values using a number close to the lowest value of the range of proteins plus or minus the standard deviation. After data imputation these proteins were included in subsequent statistical analysis. The Blast2GO software was applied to determine gene ontology terms (GO terms) relating to biological processes (BP) and molecular function (MF).

### 2.10. Statistical Analysis

Results presented in this paper are the mean of at least two independent determinations and the results are presented as the mean ± standard error. Experimental data were tested for statistical significance using a Student’s t-test. For all experimentation a p-value of ≤0.05 was considered statistically significant All the statistical analyses listed were performed using GraphPad Prism. 

### 2.11. Data Availability

The MS proteomics data and MaxQuant search output files have been deposited to the ProteomeXchange Consortium [25] via the PRIDE partner repository with the dataset identifier PXD015254. 

## 3. Results

### 3.1. Effect of Atorvastatin on Growth of A. fumigatus

**Figure 1:**
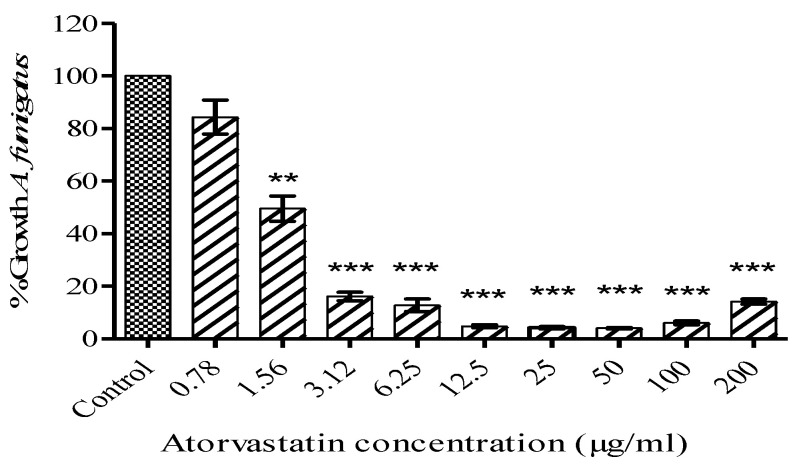
Effect of atorvastatin on growth of *A fumigatus*. *A. fumigatus* conidia (initial concentration 1 × 10^6^/mL) were exposed to atorvastatin in SAB culture medium. Growth (%) was calculated by comparing atorvastatin-treated *A. fumigatus* to control cells after 48 h growth (** p < 0.01; *** p < 0.001).

Exposure of *A. fumigatus* conidia (initial density 1 × 10^6^/mL) to atorvastatin for 48 h at 37 °C resulted in reduced growth. An atorvastatin dose of 6.25 µg/mL inhibited growth by 86.4% ± 2.7% (p < 0.0001) while doses of 3.12 and 1.56 µg/mL reduced growth by 83% ± 2% and 47.6% ± 4.7% (p < 0.002), respectively, at the same timepoint (Figure 1). Exposure of cultures of *A. fumigatus* to atorvastatin lead to a significant inhibition in biomass accumulation at 72 h. Cultures were pre-grown for 24 h and then supplemented with atorvastatin at the concentrations stated for a further 48 h. Biomass was reduced from 3.45 ± 0.13 g/100 mL in control cultures to 2.28 ± 0.08 g/100 mL (p = 0.0018) at an atorvastatin concentration of 1.5 µg/mL and at a concentration of 6 µg/mL biomass was reduced to 0.488 ± 0.04 g/100 mL (p < 0.0001) (Figure S1). Incorporation of atorvastatin into malt extract agar also led to a reduction in growth of *A. fumigatus* colonies (Figure S2).

### 3.2. Measurement of Ergosterol from A. fumigatus Mycelium Exposed to Atorvastatin

**Figure 2:**
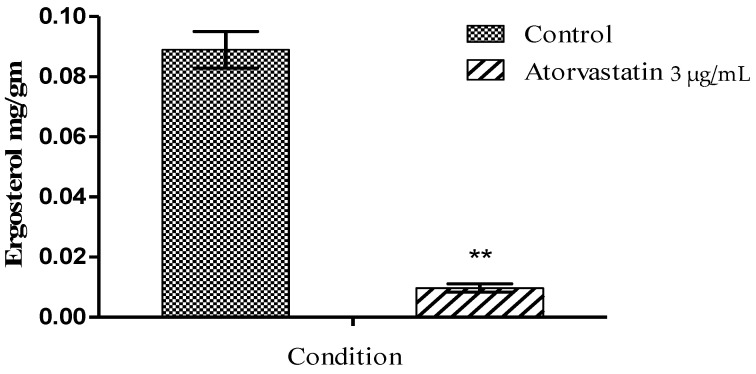
Atorvastatin (3 µg/mL) exposure reduced the ergosterol content of *A. fumigatus*. Ergosterol quantification was performed using a gas chromatograph with a flame ionization detector and a chrompack capillary column. Ergosterol standards were used to calibrate the instrument. Ergosterol content was expressed in terms of mg/mL (** p < 0.01).

Ergosterol was extracted from mycelial exposed to atorvastatin as described and quantified by GC analysis. Exposure of *A. fumigatus* cultures to atorvastatin (3 µg/mL) for 72 h lead to a significant reduction in ergosterol production (Figure 2) (control = 0.088 ± 0.008 mg/g and atorvastatin-treated mycelia = 0.006 ± 0.0012 mg/g (p < 0.01).

### 3.3. Atorvastatin Induces Leakage from A. fumigatus

**Figure 3:**
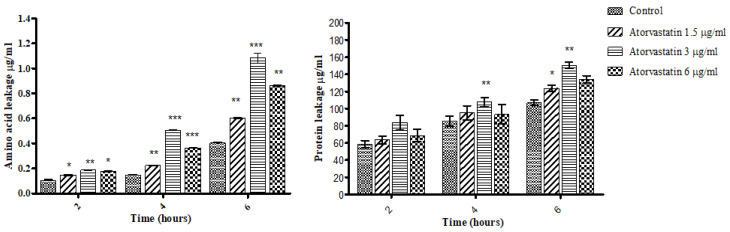
Effect of atorvastatin on the release of amino acids (a) and protein (b) from *A. fumigatus*. The effect of atorvastatin (3 μg/mL) on *A. fumigatus* amino acid and protein release was determined by a ninhydrin colorimetric assay and Bradford protein assay, respectively. Atorvastatin treatment resulted in increased amino acid and protein release relative to control cells (* p < 0.05; ** p < 0.01; *** p < 0.001).

The effect of atorvastatin on membrane permeability was assessed by quantifying amino acid, protein and gliotoxin release from cells. Exposure of *A. fumigatus* hyphae to atorvastatin (3 µg/mL) for 4 or 6 h led to an increase in amino acid release from cells (Figure 3A). At 4 h amino acid release from control cells was 0.14 ± 0.0035 µg/mL but in the 3 µg/mL atorvastatin treatment it was 0.5 ± 0.0037 µg/mL (p < 0.0001). At 6 h exposure the amino acid release from control cells was 0.4 ± 0.004 µg/mL and 1.08 ± 0.04 µg/mL (p < 0.0001) from the cells supplemented with the 3 µg/mL atorvastatin. Exposure of *A. fumigatus* to atorvastatin also led to increased protein leakage (Figure 3B). Exposure to atorvastatin for 4 h lead to a protein concentration of 107.6 ± 5.2 µg/mL in the 3 µg/mL treatment compared to 85.6 ± 6 µg/mL in the control, and after 6 h in control the protein release was 106.8 ± 3.1 µg/mL compared to 150.5 ± 3.6 µg/mL in the treatment (3 µg/mL) (p < 0.02).

**Figure 4:**
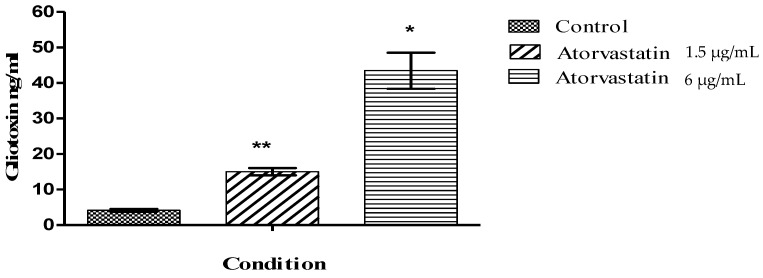
Atorvastatin exposure increased the gliotoxin release from *A. fumigatus*. Gliotoxin quantification was performed using an HPLC. The gliotoxin standards were used to calibrate the instrument. Gliotoxin content was expressed in terms of ng/mL (* p < 0.01; ** p < 0.01).

Atorvastatin exposure also induced an increase in the release of gliotoxin from *A. fumigatus* cultures (Figure 4). After 72 h growth at 37 °C gliotoxin was 14.04 ± 1.01 ng/mL at an atorvastatin concentration of 1.5 µg/mL compared to the control, which was 4.2 ± 0.6 ng/mL (p = 0.005). At an atorvastatin concentration of 6 µg/mL the gliotoxin concentration was 47.22 ± 5.06 ng/mL (p = 0.01).

### 3.4. Proteomic Analysis of Response of A. fumigatus to Atorvastatin

**Figure 5:**
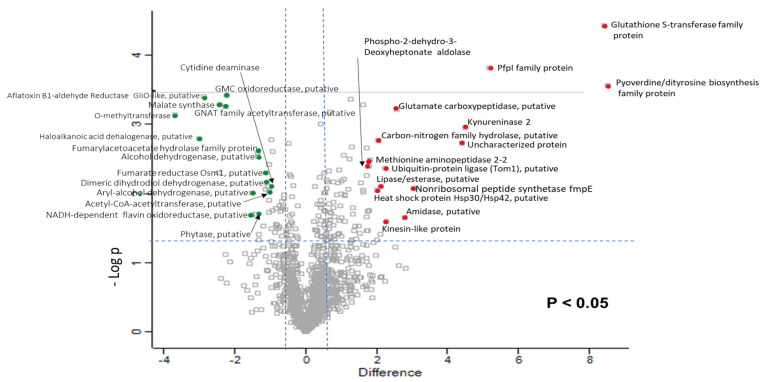
Volcano plot showing the distribution of quantified proteins according to p value (−log10 p-value) and fold change (log2 mean LFQ intensity difference). Proteins above the horizontal line are considered statistically significant (p value < 0.05) and those to the right and left of the vertical lines indicate relative fold changes of ±1.5.

Proteomic analysis of *A. fumigatus* cultures exposed to atorvastatin revealed 25,915 peptides representing 1360 proteins, and 128 proteins were determined to be differently abundant with a fold change of >1.5. In total 83 proteins were increased in abundance and 45 were decreased in abundance. These proteins were subsequently used to statistically analyse the total differentially expressed group after imputation of the zero values as described and were then included in statistical analysis after data imputation. A principal component analysis (PCA) was performed on all filtered proteins and clearly distinguished the control and atorvastatin treated *A. fumigatus* samples (Figure S3). The volcano plot shows the relative distribution of these proteins (Figure 5), and of the proteins that increased in abundance following exposure to atorvastatin, the most notable were isocyanide synthase A (+8.52-fold), glutathione S-transferase family protein (+8.43-fold), kynureninase 2 (+4.48-fold), and non-ribosomal peptide synthetase fmpE (+3.06-fold). The main peptides decreased in abundance were O-methyltransferase (-3.68-fold), allergen Asp f 15 (-2.45-fold) and GNAT family acetyltransferase (-2.25-fold).

The main enzyme classes that were altered in abundance in *A. fumigatus* treated with atorvastatin were oxidoreductases, transferases and hydrolases (Figure S4). Oxidoreductase enzymes that increased in abundance in atorvastatin treatment of *A fumigatus* were pyoverdine/dityrosine biosynthesis family protein (+8.52-fold), 5-demethoxyubiquinone hydroxylase, mitochondrial (+1.73-fold), alcohol dehydrogenase, putative (+1.6-fold) and Psi-producing oxygenase A (+1.55-fold). Thirteen oxidoreductase enzymes showed a decrease in abundance and included aflatoxin B1-aldehyde reductase GliO-like, putative (−2.86-fold), aryl-alcohol dehydrogenase, putative (−1.52-fold) and NADH-dependent flavin oxidoreductase, putative (−1.50-fold). Enzymes with transferase activity increased in abundance included glutathione S-transferase family protein (+8.43-fold), phosphatidylinositol transporter, putative (+2.00-fold) and allergen Asp f 4 (+1.42-fold), while O-methyltransferase (−3.68-fold), malate synthase (−2.40-fold) and GNAT family acetyltransferase, putative (−2.25-fold) were decreased in abundance. Nine hydrolytic enzymes were increased in abundance and these included kynureninase (4.48-fold), amidase putative (+2.77-fold), lipase/esterase putative (+2.12-fold) and methionine aminopeptidase 2-2 (+1.83-fold). In contrast two hydrolytic enzymes were reduced in abundance (haloalkanoic acid dehalogenase, putative (−2.99-fold) and phytase, putative (−1.42-fold).

## 4. Discussion

Statins are widely used for the control of cholesterol and function by inhibiting the action of 3-hydroxy-3-methylglutaryl-CoA (HMG-CoA) reductase [26,27]. Patients on statin therapy display lower incidences of fungal infections [19,28], prompting the suggestion that statins may display a significant antifungal effect [20] and could have applications in treating recalcitrant fungal infections. Statins can inhibit the growth of *C. albicans* [15] and growth inhibition may be via a reduction in ergosterol content [29]. High dose of statin treatment has been correlated with immune-modulatory effects in some patients [30,31] and, in particular, the induction of modulation in the activity of regulatory T cells [32].

Exposure of *A. fumigatus* to atorvastatin reduced fungal growth and induced increased membrane permeability as evidenced by increased release of protein and amino acids. Maximum amino acid and protein leakage occurred in the 3 μg/mL treatment at 6 h and this concentration may be optimal for this effect. Statins inhibit the production of cholesterol in mammals and have previously been shown to inhibit ergosterol biosynthesis in fungi [29]. Reduced ergosterol in atorvastatin-treated *A. fumigatus* cells may alter the integrity of the membrane and thus lead to increased permeability. Cultures treated with atorvastatin also released elevated levels of gliotoxin, which also has been observed in *A. fumigatus* exposed to amphotericin B and may indicate an oxidative stress response [33].

Proteomic analysis revealed an increased abundance of a range of proteins involved in dealing with oxidative stress, including pyoverdine/dityrosine biosynthesis (+8.52-fold), 5-demethoxyubiquinone hydroxylase, mitochondrial (+1.74-fold) and alcohol dehydrogenase (+1.61-fold). In *Saccharomyces cerevisiae*, dityrosine is a major component of the spore wall surface and plays a role in the resistance of ascospores to environmental stress [34]. Furthermore, the glutathione S-transferase family protein is increased in abundance (+8.43-fold), and this protein functions to detoxify xenobiotics. The fumipyrrole biosynthetic (fmpE) protein was also increased in abundance (+3.06-fold). Deletion of fmpE in *A. fumigatus* resulted in reduced growth and sporulation of the mutant strains but virulence was not altered as compared to the WT strain in a murine infection model [35].

A range of heat shock proteins, such as Hsp30/Hsp42 (+2.02-fold), also increased in abundance in response to atorvastatin. Heat shock proteins play an important role in adaptation of the fungal cell to environmental and chemical stresses [36]. *A. fumigatus* heat shock proteins are also immunodominant and elicit strong humoral immune responses [37]. Proteomic analysis also revealed the reduced abundance of a number of peptides associated with virulence, including aflatoxin B1 (−2.86-fold) and allergen Asp f 15 (−2.45-fold). Asp f 15 is cell-wall associated and its decreased abundance could be due to an altered cell-wall composition as a result of an altered membrane composition.

Statins inhibit the growth of *A. fumigatus* and supplementation of media with ergosterol or cholesterol blocked the growth-inhibiting effect of atorvastatin, thus indicating the specificity of statins for the mevalonate synthesis pathway [15]. However, another study found that statins were fungistatic against *A. fumigatus*, but the MICs were greater than the clinically achievable concentrations [16]. Furthermore, a variety of *Candida* strains that were resistant to fluconazole and nystatin were all sensitive to atorvastatin [38]. Atorvastatin combined with fluconazole improved cryptococcosis caused by *Cryptococcus gattii* and mice treated with atorvastatin had increased survival and better clinical outcomes; this was mediated via modifications in the capsule and cell membrane in the presence of a statin that induced cell death within phagocytes [39].

## 5. Conclusions

The results presented here confirm previous findings obtained using statins (e.g., a reduction in ergosterol content); however, the alteration in membrane permeability caused by atorvastatin and the altered proteomic response are indicative of oxidative stress in the cells. Fully understanding the antifungal mode of action of statins may highlight novel ways to utilise these widely used drugs for the control of recalcitrant fungal infections.

## Supplementary Materials

The following are available online at https://www.mdpi.com/2309-608X/6/2/42/s1, Figure S1. The biomass of *A. fumigatus* exposed to atorvastatin (1.5 and 6 µg/mL). Figure S2: Growth area of *A. fumigatus* on MEA plates supplemented with atorvastatin. Figure S3. Principal component analysis of control and atorvastatin treated *A. fumigatus* at 24 h showing a clear distinction between control and treatment. Figure S4. Bar chart showing changes to number of proteins involved in enzyme classes at Level 3 ontology. Proteins were assigned groups based on involvement in biological processes for control and atorvastatin treated.
