# Supplementary material for: Exposure of Aspergillus fumigatus to Atorvastatin Leads to Altered Membrane Permeability and Induction of an Oxidative Stress Response
Source: J Fungi (Basel). 2020 Mar 26;6(2):42. doi: 10.3390/jof6020042 (PMC7344724; doi:10.3390/jof6020042)
Supplement: Supplementary file 1 [file jof-06-00042-s001.pdf]

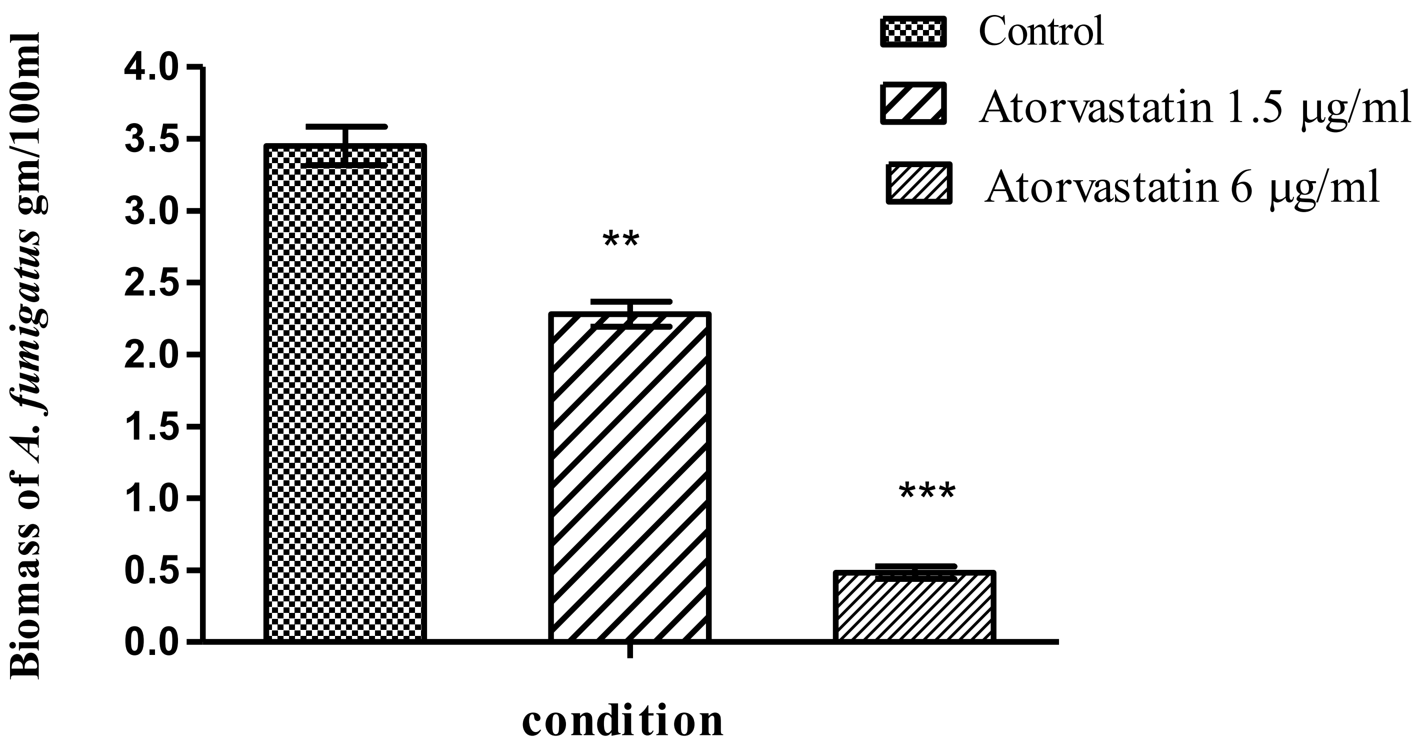

Fig. S1. The biomass of *A. fumigatus* exposed to atorvastatin (1.5 and 6 µg/ml). *A. fumigatus* was grown in SAB culture medium at 37^{0}C. Biomass was measured after 72 h growth (**; $p < 0.01$ ***; $p < 0.01$).

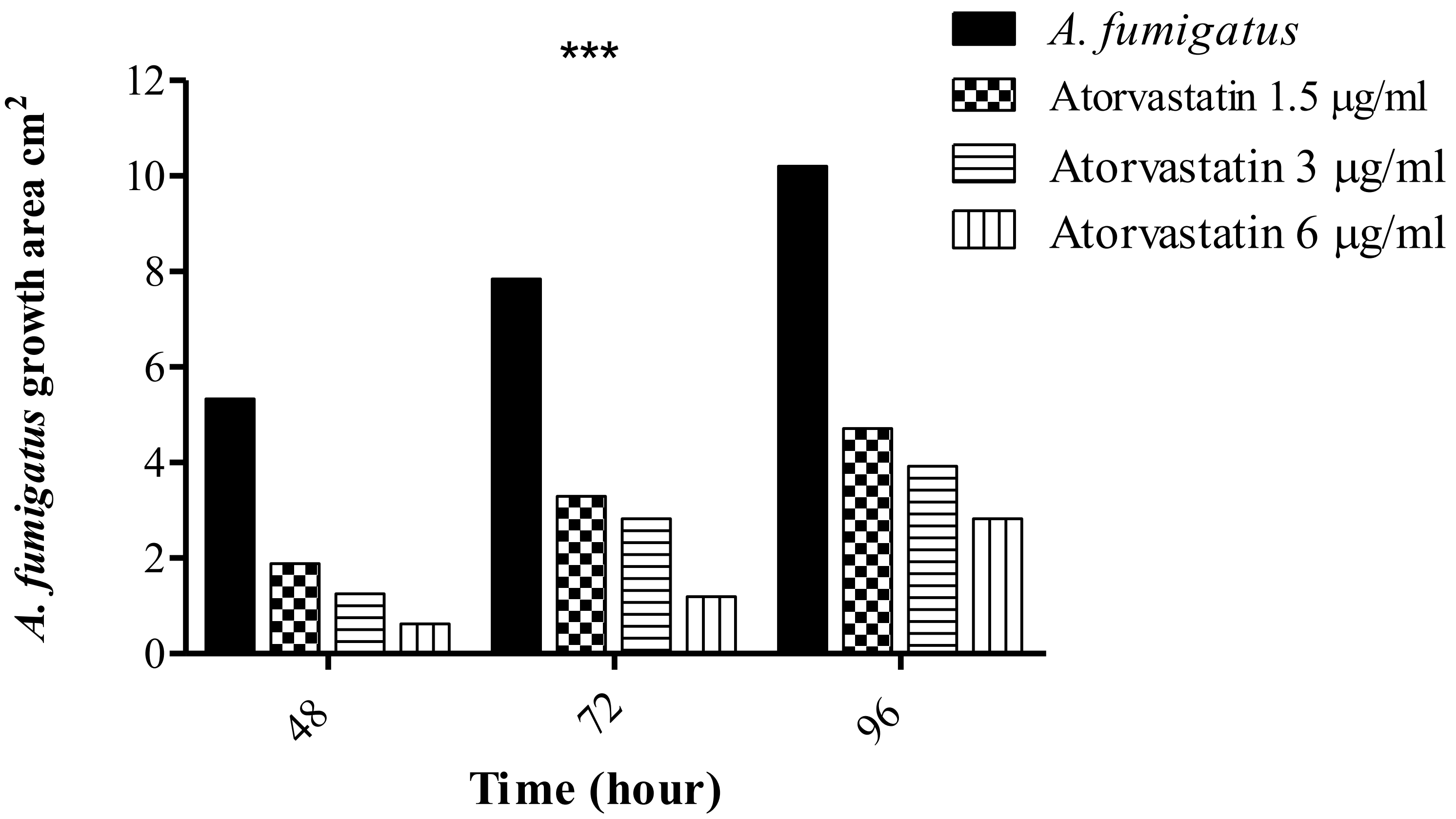

Fig. S2: Growth area of *A. fumigatus* on MEA plates supplemented with atorvastatin (1.5, 3 and 6 µg/ml) vs time (48, 72 and 96 hour) (***; $p < 0.001$).

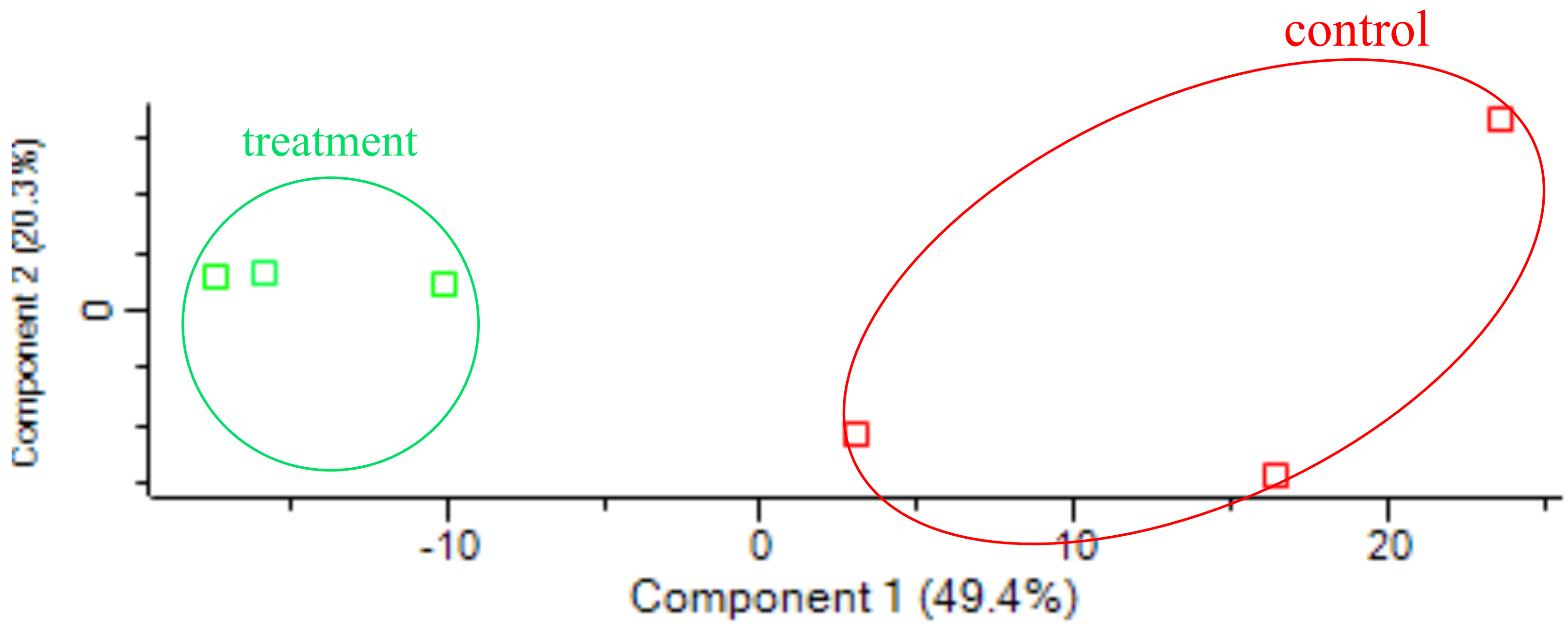

Fig S3. Principal component analysis of control and atorvastatin treated *A. fumigatus* at 24 h showing a clear distinction between control and treatment.

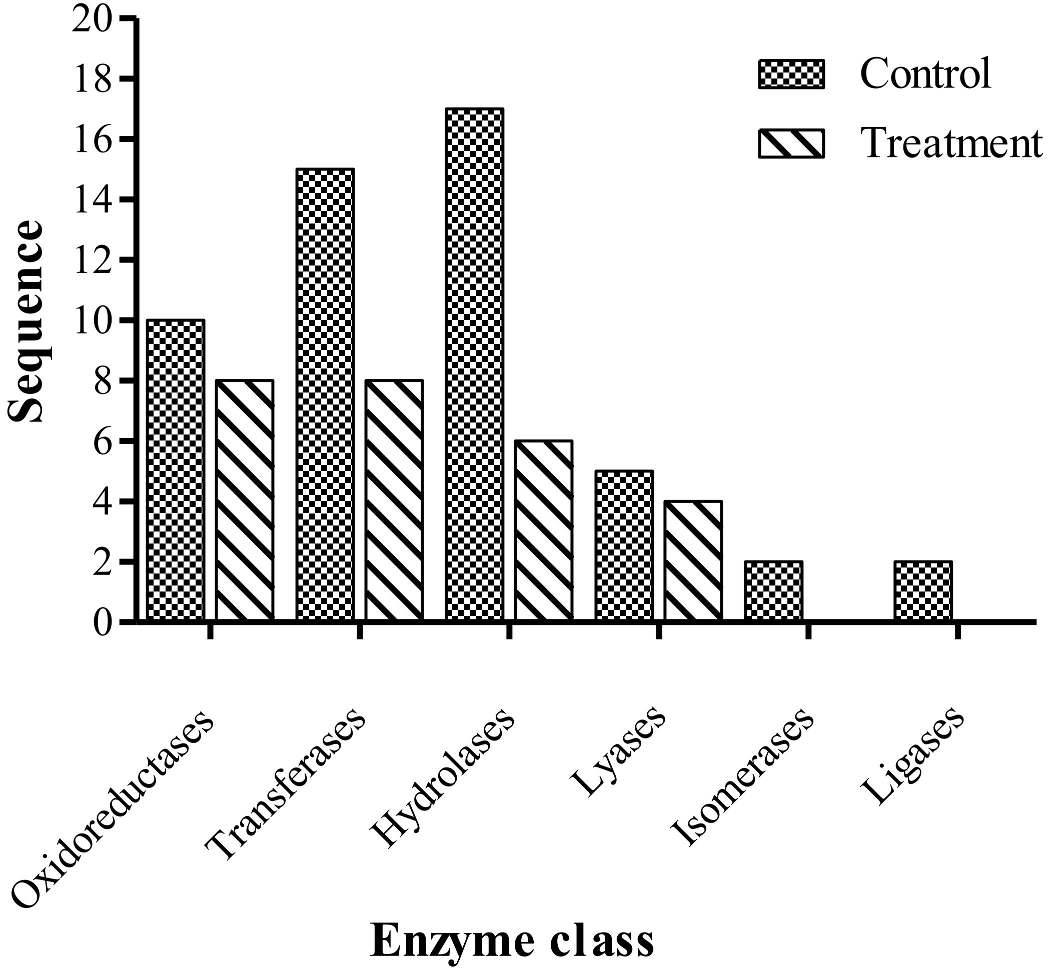

Fig. S4. Bar chart showing changes to number of proteins involved in enzyme classes at level 3 ontology. Proteins were assigned groups based on involvement in biological processes for control and atorvastatin treated. Open bar: control, closed bar: treated.
